# Liraglutide Therapy for Type 2 Diabetes: Overcoming Unmet Needs

**DOI:** 10.3390/ph3030764

**Published:** 2010-03-22

**Authors:** Åke Sjöholm

**Affiliations:** Karolinska Institutet, Department of Clinical Science and Education, Division of Internal Medicine, Unit for Diabetes Research, Södersjukhuset, SE-118 83 Stockholm, Sweden; Email: ake.sjoholm@sodersjukhuset.se; Tel.: +46-861-63-219; Fax: +46-861-63-146/2933.

**Keywords:** GLP-1, liraglutide, LEAD, glycaemic control, type 2 diabetes

## Abstract

Although advances have been achieved in the management of type 2 diabetes, current treatment options for patients with this disease still fail to address disease progression, glycaemic control remains suboptimal and therapies are often associated with weight gain and hypoglycaemia. Thus, new antidiabetes therapies are being sought. Glucagon-like peptide-1 (GLP-1) and glucose-dependent insulinotropic polypeptide (GIP) are incretin hormones that have been the recent focus of research. The physiological action of GLP-1, in particular, has demonstrated its potential in addressing the therapeutic needs of patients with type 2 diabetes. To exploit this action, liraglutide, a human GLP-1 analogue that shares 97% of its amino acid sequence identity with native GLP-1, has been developed. In a recent phase 3 trial programme (LEAD, Liraglutide Effect and Action in Diabetes), treatment with liraglutide was associated with substantial improvements in glycaemic control and low risk of hypoglycaemia. In addition, reductions in weight and systolic blood pressure were reported. There is also an indication that liraglutide is capable of improving β-cell function and increasing β-cell mass. Thus, liraglutide may overcome the limitations with current therapies and help to address the unmet clinical needs of patients with type 2 diabetes.

## 1. Introduction

Diabetes is a progressive disease characterised by impaired β-cell function, and reduced insulin sensitivity and secretion. Over time, glycaemic control deteriorates and exacerbates the risk of patients experiencing micro- and macrovascular complications [1].

**Figure 1:**
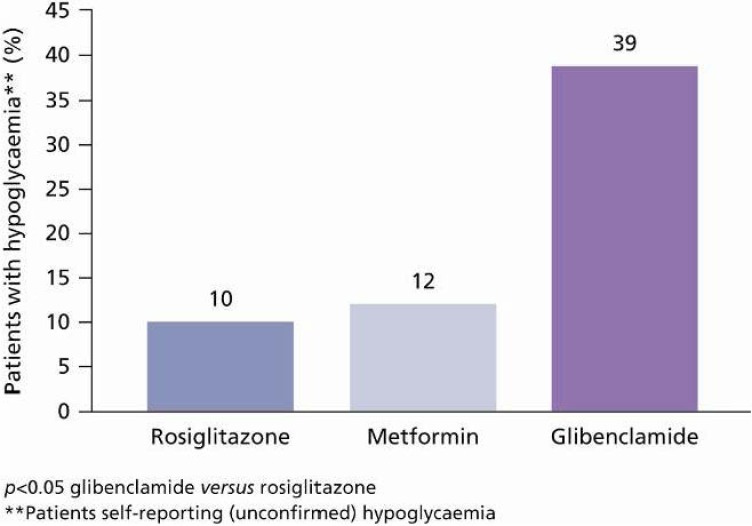
Hypoglycaemia as Shown in ADOPT (A Diabetes Outcome Progression Trial) [2].

**Figure 2:**
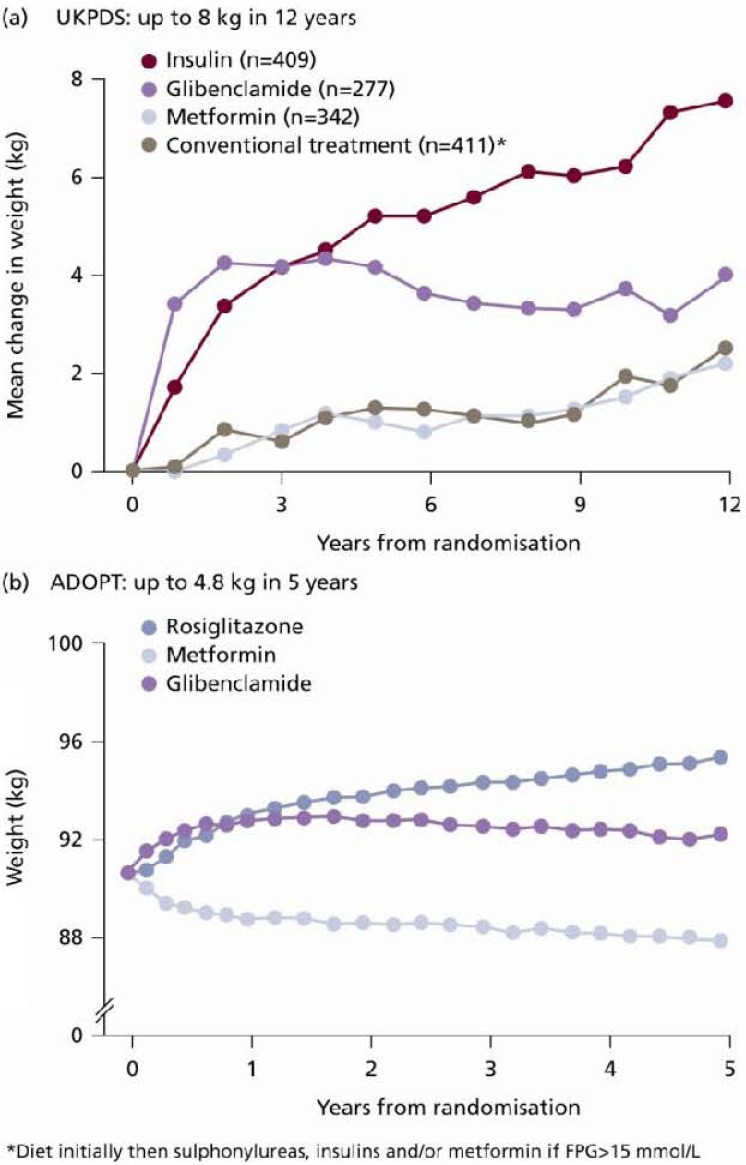
Weight change associated with each treatment group included in the (a) UKPDS and (b) ADOPT study [1,2]. Figure 2a reprinted from [1] with permission from Elsevier (© 1998); Figure 2b reprinted from with [2] permission from the Massachusetts Medical Society [2] (© 2006). All rights reserved.

Although there is an abundance of treatment options and guidelines available for the management of type 2 diabetes, they are unable to avert the natural progression of the disease and sustain glycaemic control in the long term. Furthermore, currently available treatment options (such as sulphonylureas [SUs], thiazolidinediones [TZDs] and most insulins) are often associated with hypoglycaemia and weight gain [1,2] (Figure 1 and Figure 2). Such adverse effects can have detrimental health implications for the patient. For example, hypoglycaemia can be an unpleasant side effect of antidiabetes therapy that can compromise patient adherence to treatment, and serious hypoglycaemic events, left untreated, can lead to a loss of consciousness, brain damage or even death. Moreover, obesity is a common comorbidity in subjects with type 2 diabetes (affecting 60–90% of patients diagnosed). Additional weight gain as a consequence of treatment with SUs or insulin, for example, can reduce patients’ quality of life and hinder adherence to treatment. Obesity is also an independent risk factor for cardiovascular (CV) disease, further compromising patient outcomes [3,4,5].

The greatest challenge in treating patients with type 2 diabetes is optimising therapy to address the current unmet needs, which include:

- improving glycaemic control without compromising safety, *e.g.*, hypoglycaemia
- preserving β-cell function
- providing clinically meaningful weight loss
- addressing cardiovascular risk factors accompanying diabetes
- offering a simple and flexible regimen

New and effective treatment options are being sought to address the unmet clinical needs of patients with type 2 diabetes. Indeed, therapies are emerging that target the incretin system by exploiting the physiological actions of glucagon-like peptide-1 (GLP-1) and glucose-dependent insulinotropic polypeptide (GIP). These therapies may provide solutions to all of these challenges.

## 2. Background to the Incretin Effect

**Figure 3:**
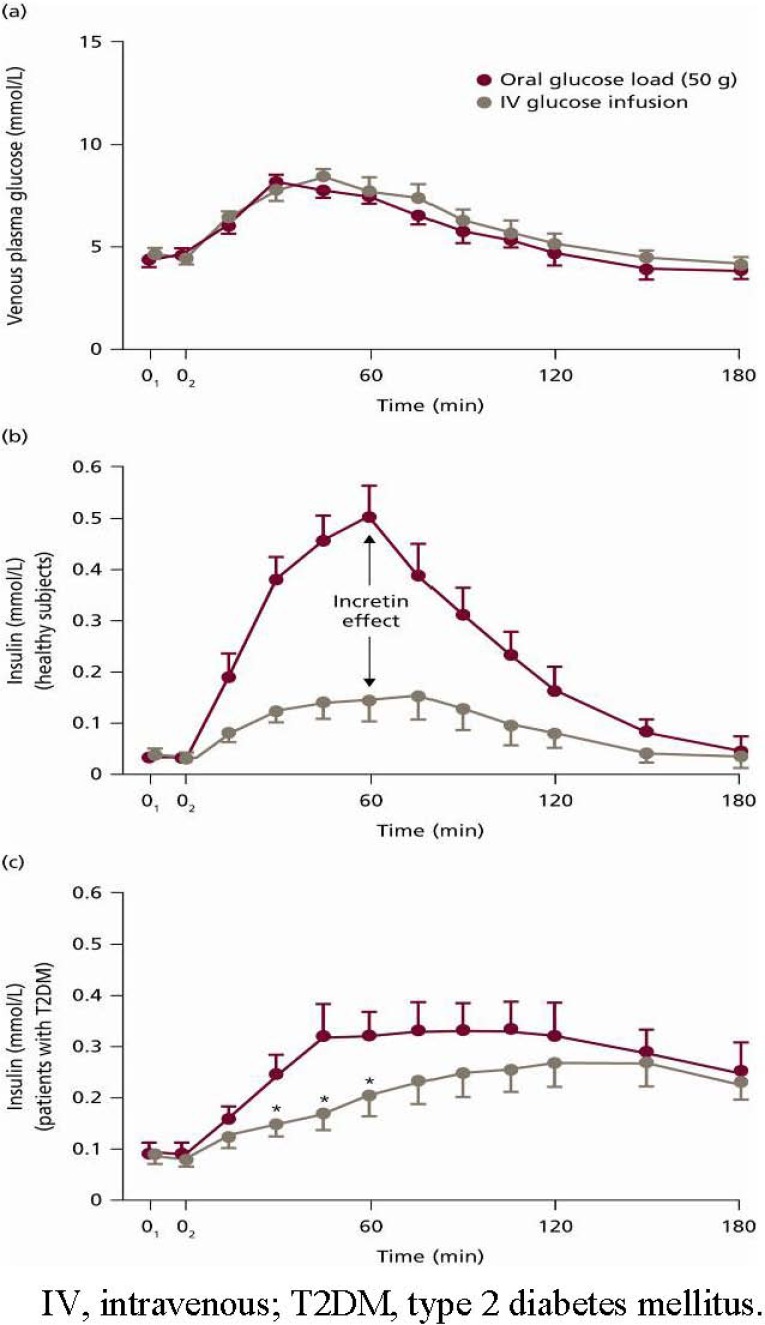
(a) Plasma Glucose and Venous Insulin Response in the Plasma; (b) Normal Insulin Response to Oral/Intravenous Glucose Load; (c) This Response in People with Type 2 Diabetes [7]. Reprinted with permission from Springer, ^© ^1986.

GLP-1 and GIP are gut-derived hormones known as ‘incretins’. The incretin hormones have multiple physiological actions; most importantly, they play a crucial role in glucose homeostasis. The action of the incretin hormones accounts for 50–70% of insulin secretion after oral glucose intake [6]. This ‘incretin effect’ is characterised by the more pronounced plasma insulin secretion observed with oral *versus* intravenous glucose administration despite matching glucose profiles [6] (Figure 3a and Figure 3b).

The incretin effect is blunted in subjects with type 2 diabetes [7] (Figure 3c). In such patients, GLP-1 infusion (but not GIP) markedly improves both the early and late phases of insulin secretion in response to glucose [8,9]. As such, it is GLP-1 rather than GIP that has been the focus of research as a promising treatment for type 2 diabetes.

## 3. GLP-1 and its Relevance to Treating Unmet Needs in Type 2 Diabetes

The action of GLP-1 on β-cell receptors enhances insulin secretion in a glucose-dependant manner [10], which, in turn, minimises the risk of hypoglycaemia. In addition, animal studies have indicated that GLP-1 has the ability to preserve β-cell function by suppressing β-cell apoptosis and stimulating neogenesis and proliferation [11,12].

**Figure 4:**
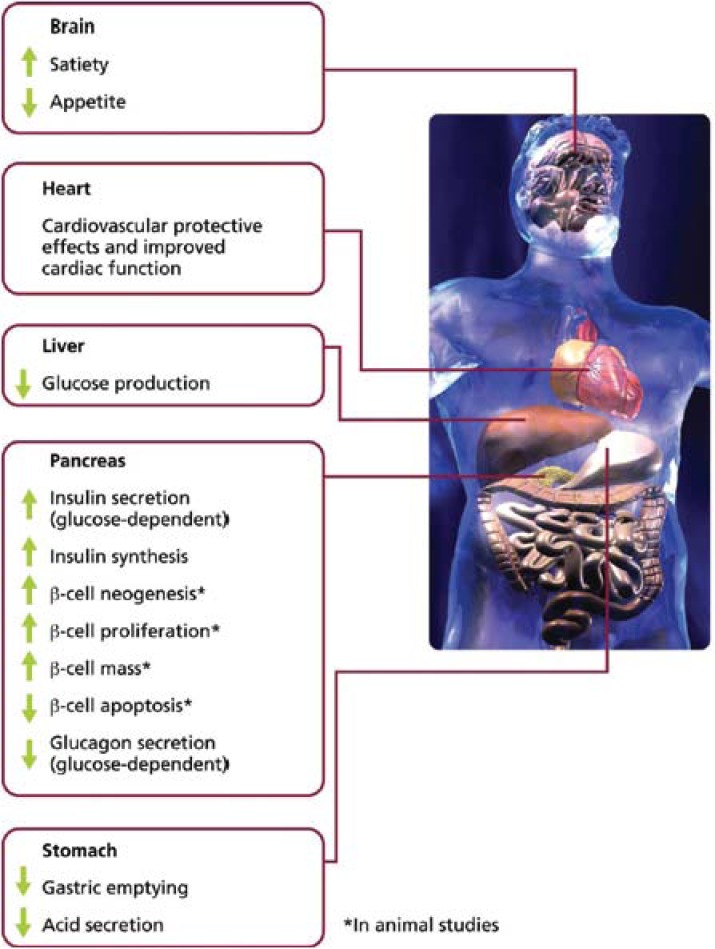
Physiological Pathways of GLP-1.

Other clinical advantages associated with GLP-1 may be explained by its hormonal influences on the gastrointestinal, central nervous system and CV systems (Figure 4). GLP-1 has demonstrated the ability to slow gastric emptying and suppress appetite, resulting in satiety and weight loss [10,13]. 

GLP-1 also appears to exert a protective effect on the myocardium, particularly in ischaemic conditions [14,15]. For example, Bose and colleagues reported that an intravenous infusion of GLP-1 in rats prior to induced ischaemia significantly reduced myocardial infarction compared with saline [16]. Studies have also reported improved myocardial function in patients with type 2 diabetes and congestive heart failure [17], and infusion of recombinant GLP-1 has been shown to improve left ventricular function in patients with acute myocardial infarction after primary angioplasty [18]. Improvement in endothelial function has also been reported with GLP-1: a significant increase in brachial artery diameter was observed in patients with type 2 diabetes [19]. 

Furthermore, GLP-1 has been found to reduce systolic blood pressure (SBP). This may be, in part, due to its ability to increase diuresis and natriuresis, thus reducing blood volume and central venous pressure. This is an important observation since CV disease is a common comorbidity of type 2 diabetes, and a mean reduction in SBP of 5.6 mmHg has been shown to reduce mortality from CV disease by 18% [20].

## 4. Clinical Limitations of GLP-1 Due to the Presence of DPP-4

As active GLP-1 is secreted from L-cells in the distal small intestine, it is rapidly degraded by the enzyme dipeptidyl peptidase IV (DPP-4). An intravenous bolus of GLP-1 has a half-life of 1.5–2.1 min [21], meaning that a constant infusion of GLP-1 is required to provide any therapeutic value.

## 5. Development of Liraglutide

**Figure 5:**
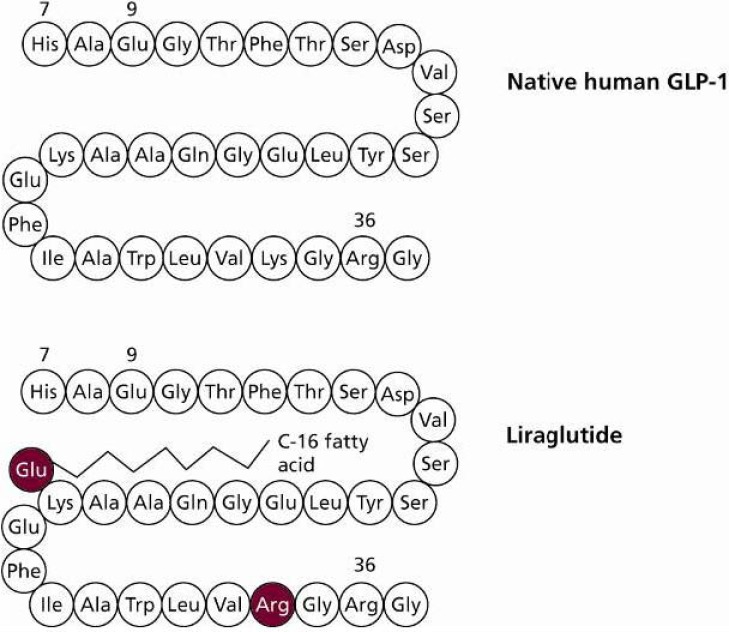
Amino Acid Structure of Native Human GLP-1 and Liraglutide.

In order to overcome the short half-life of native GLP-1, longer-acting GLP-1 agents such as liraglutide have been developed. Liraglutide is an analogue of native human GLP-1, in which Lys34 has been substituted with Arg34 at the N-terminal and a fatty acid chain added to Lys26 [21] (Figure 5). These modifications mean that liraglutide shares 97% amino acid identity with native human GLP-1, whereas exenatide, a GLP-1 mimetic, shares just 53% sequence identity at the N-terminal 30-aminoacid peptide, with no mammalian homology for the remaining nine amino acids.

Liraglutide’s fatty acid side chain allows it to self-associate and form heptamers. This allows liraglutide to be absorbed slowly via the subcutaneous route [21]. Maximum plasma concentration levels are achieved between 9–12 hours after dosing [22].The fatty acid moiety, which is internally orientated when liraglutide is heptameric, also allows liraglutide in monomeric form to bind to serum albumin in the bloodstream [23] and resist DPP-4 degradation, resulting in a half-life of approximately 13 hours [24]. These properties make liraglutide suitable for once-daily dosing [24]. 

## 6. Clinical Effects of Liraglutide

The phase 3 clinical development programme for liraglutide, LEAD (Liraglutide Effect and Action in Diabetes) is the largest clinical development programme ever conducted by Novo Nordisk in diabetes; a total of 4,456 subjects were included, recruited at more than 600 sites across 40 countries, of which 2,739 patients were treated with liraglutide. LEAD included six trials and was designed to investigate the efficacy and safety of patients treated with liraglutide across the continuum of care of type 2 diabetes *versus* placebo. 

**Figure 6:**
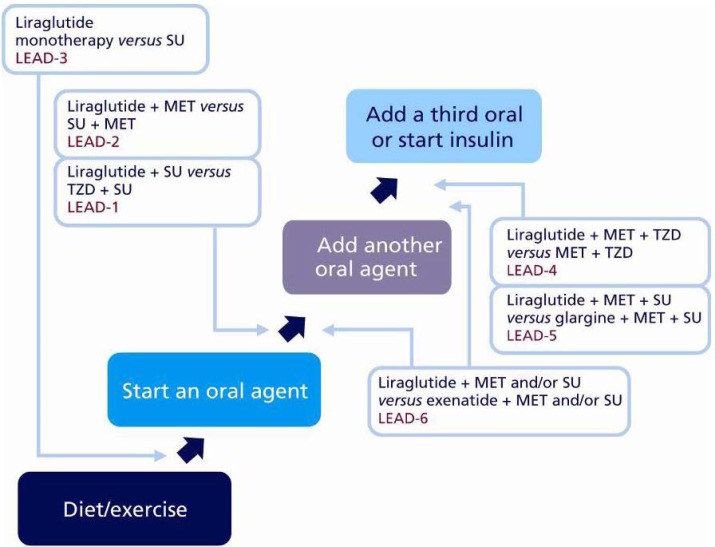
Liraglutide Implementation Throughout the Continuum of Care.

In the LEAD trials, liraglutide was also compared to some commonly used antidiabetic therapies. In addition to the registration studies (LEAD-1 to LEAD-5), a head-to-head trial against exenatide (LEAD-6) was also completed (Figure 6). 

### 6.1. HbA_1c_, FPG and PPG

**Figure 7:**
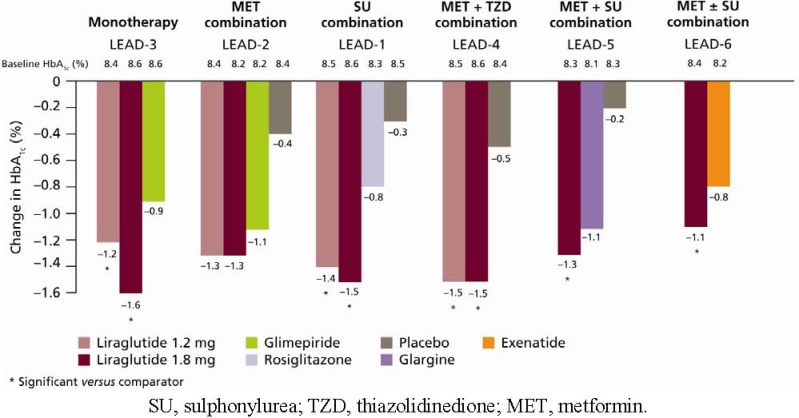
Change in HbA_1c_ from Baseline (LEAD-1–6) for Overall Population (LEAD-4–6), Add-on to Diet and Exercise (LEAD-3) and Add-on to Previous OAD Monotherapy (LEAD-1–2) [25,26,27,28,29,30].

**Figure 8:**
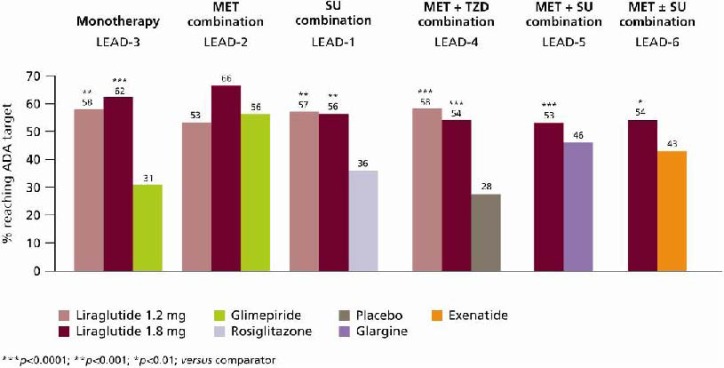
Percentage of Subjects Reaching ADA Target HbA_1c_ <7.0% in the LEAD-1–6 Trials [25,26,27,28,29,30].

The LEAD programme demonstrated that liraglutide, used as monotherapy or in combination with one or two oral antidiabetic drugs (OADs), provides substantial reductions in HbA_1c_. Liraglutide reduced HbA_1c_ levels to a significantly greater extent than its active comparators, except in LEAD-2, where HbA_1c_ reductions with liraglutide were comparable to glimepiride plus metformin (−1.0%) in the overall study population. In the LEAD-2 study, more substantial reductions were demonstrated in the sub-group of patients receiving liraglutide 1.2 mg and 1.8 mg as an add-on to previous OAD therapy compared with glimepiride plus metformin, though not to a significant extent (both -1.3% *vs*. 1.1%, respectively; p = NS) (Figure 7) [26]. Across the LEAD trials, reductions in HbA_1c_ of up to 1.6% were achieved with the higher doses of liraglutide (1.2 mg and 1.8 mg) relative to baseline (Figure 7) [25,26,27,28,29,30]. Reductions in HbA_1c_ primarily occurred within 8–12 weeks when liraglutide 1.2 mg and 1.8 mg were added to metformin (LEAD-2) [26], glimepiride (LEAD-1) [25] and metformin plus rosiglitazone (LEAD-4) [28]; these reductions were sustained throughout each study period and were significantly greater compared with placebo (Figure 7). A higher percentage of subjects in the liraglutide-treated groups reached American Diabetes Association (ADA) target HbA_1c_ < 7.0% in all of the LEAD studies compared with active comparators (Figure 8) [25,26,27,28,29,30].

The greatest reduction in HbA_1c_ (−1.60%) was experienced in the LEAD-3 trial (liraglutide monotherapy) by the subgroup of patients previously on diet and exercise: the true initial monotherapy population [27]. In the head-to-head study of liraglutide 1.8 mg once daily *versus* exenatide 10 μg twice daily (as add-on to metformin and/or SU therapy), mean HbA_1c_ reduction was significantly greater with liraglutide treatment than with exenatide (–1.12% *versus* –0.79%, *p* < 0.0001), and corresponded to more patients achieving HbA_1c_ <7.0% (54% *versus* 43%, respectively; odds ratio 2.02; 95% confidence interval [CI] 1.31 to 3.11; *p* = 0.0015) [30] (Figure 8).

**Figure 9:**
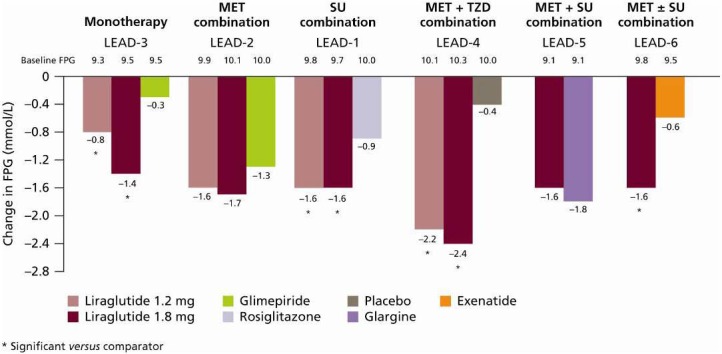
Change in FPG across the LEAD-1–6 Studies [25,26,27,28,29,30].

Liraglutide also provided substantial reductions in fasting plasma glucose (FPG) across the continuum of care. FPG reductions of up to −2.4 mmol/L were reported with liraglutide across the LEAD-1–6 studies [25,26,27,28,29,30] (Figure 9).

Liraglutide was also shown to be effective at reducing postprandial glucose (PPG); consistent reductions were observed in peak PPG (across all three meals) in the LEAD-1–5 studies [25,26,27,28,29]. In the LEAD-6 study, there was a numerically greater reduction in mean PPG after lunch with liraglutide compared with exenatide (2.74 *versus* 2.35; not significant). However, exenatide is given twice daily, before morning and evening meals, thus PPG was reduced more with exenatide *versus* liraglutide during these peak times [30].

### 6.2. β-cell Function

The LEAD trials have reported increases in β-cell function (as measured by HOMA-B) of 28–34% from baseline after liraglutide treatment [25,26,27,28,29,30]. β-cell function increased more substantially with liraglutide treatment compared with exenatide (32.12% *versus* 2.74%, respectively; *p* < 0.0001) [30]. As GLP-1s are insulinotropic, HOMA-B is not the most ideal indicator of β-cell function, but, given the paucity of β-cell function data currently available, this measure provides some, albeit limited, insight into the potential of the drug, pending further research. A reduction in pro-insulin:insulin ratio is a marker of improved β-cell function; in the LEAD-1 study, reductions in the pro-insulin:insulin ratio with liraglutide 1.2 mg (−0.11) and 1.8 mg (−0.10) were significantly greater compared with rosiglitazone (−0.05) and placebo (−0.01; *p* < 0.05 for all comparisons) [25]. Animal studies suggest that β-cell function is preserved through suppression of apoptosis [12], stimulation of β-cell neogenesis [11] and increased proliferation [31], but these mechanisms have yet to be shown in human subjects.

### 6.3. Weight

**Figure 10:**
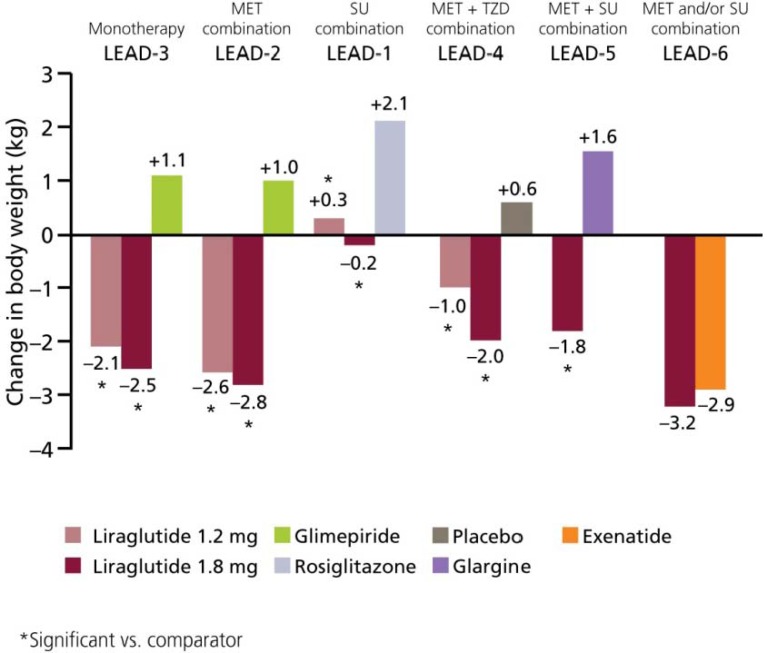
Change in Body Weight across the LEAD-1–6 Studies [25,26,27,28,29,30].

Treatment with liraglutide significantly reduced weight in the LEAD-1–6 trials (Figure 10). Liraglutide has a more positive effect on weight than active comparators [25,26,27,28,29]. Although not significant, weight reductions reported with liraglutide were more substantial compared with exenatide: –3.24 kg *versus* –2.87 kg, respectively [30]. 

Weight loss also appears to be sustained; a reduction of −2.45 kg was reported in patients treated with liraglutide 1.8 mg monotherapy (LEAD-3) [27]. The majority of this weight loss occurred primarily in the first 16 weeks and was maintained throughout the 52-week study period. In this trial, weight reduction was significantly greater with liraglutide *versus* glimepiride (+1.12 kg, *p* < 0.0001) [27]. Significant reductions in body weight were also observed when liraglutide was added to metformin (LEAD-2) [26] and metformin plus rosiglitazone (LEAD-4) [28] compared with placebo and their respective trial comparators (Figure 10; *p* < 0.0001 for both comparisons); these reductions were greater after week 8 and were maintained throughout the trial [26,28]. Of note, it was evident that weight loss was more substantial in subjects with a higher baseline body mass index. This is of particular advantage in obese subjects and those at greater risk of developing CV disease, as they would appear to experience greater weight loss than leaner patients [29].

Furthermore, an analysis of body composition (measured by dual energy X-ray absorptiometry) in subjects from the LEAD-2 and LEAD-3 study suggested that the majority of weight loss observed with liraglutide was due to a loss in fat tissue rather than lean tissue. As demonstrated by computerised tomography, visceral adipose tissue was accountable for most fat loss (13–17%), and was more substantial than the reductions in subcutaneous adipose tissue (5–9%) [32]. This is an important observation, since visceral adipose tissue has a greater association with increased lipids and glucose than subcutaneous tissue, and consequently insulin resistance. LEAD-1 studied liraglutide in combination with SUs, and here, weight loss was less significant from baseline andin the lower dose group (1.2 mg liraglutide), modest weight gain was observed. These findings are thought to be the result of SU use in this population Notably, weight change was still significant in favour of liraglutide versus the comparator (SU and rosiglitazone).

### 6.4. Systolic Blood Pressure

**Figure 11:**
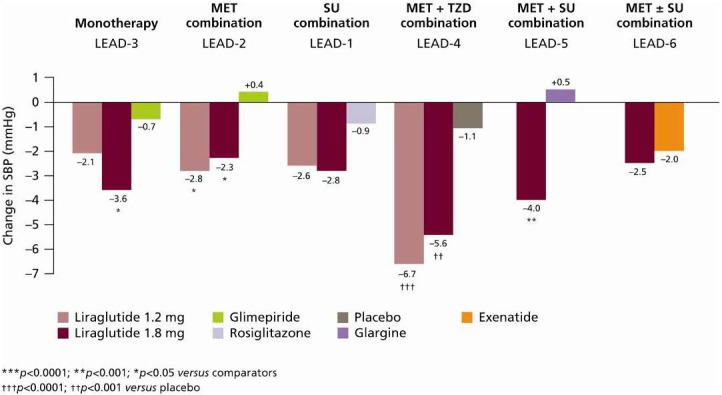
Change in SBP across LEAD-1–6 [25,26,27,28,29,30].

Liraglutide provides clinically significant reductions in SBP, as demonstrated across all of the LEAD trials [25,26,27,28,29,30] (Figure 11). 

SBP has also been shown to improve rapidly (as early as early weeks) following liraglutide initiation, and improvements were sustained [33]. For example, some fluctuations in SBP can be observed over time with liraglutide 1.8 mg in combination with metformin (LEAD-2) [26] or glimepiride (LEAD-1) [25]; however, SBP reduced significantly after 26 weeks (−2.3 mmHg and −2.8 mmHg, respectively *p* < 0.05 *versus* baseline for both comparisons). Moreover, SBP was reduced prior to significant treatment-induced weight loss. Indeed, the reductions in SBP predominantly occur in the first 2 weeks of treatment, whereas body weight tends not to be affected until week 8.

### 6.5. Safety and Tolerability

Since liraglutide acts in a glucose-dependent manner [10], the risk of hypoglycaemia is low. Throughout all of the LEAD trials major hypoglycaemia was rare; of the six cases reported, one of these patients was undergoing treatment with liraglutide 1.8 mg plus glimepiride [25] and the other five were treated with liraglutide 1.8 mg in combination with glimepiride and metformin [29]. Since hypoglycaemic risk is a recognised feature of SU treatment, we can assume that these reports are a consequence of combining liraglutide with an SU. Indeed, when liraglutide was used in combination with OADs (other than SUs) or as monotherapy, no major hypoglycaemia was observed [26,27,28,30]. Of note, in the LEAD-6 trial, no major hypoglycaemia occurred with liraglutide but there were two episodes in patients receiving exenatide in combination with an SU [30].

Minor hypoglycaemia is not uncommon with existing diabetes treatments; however, fewer minor hypoglycaemia events were reported with liraglutide treatment compared with conventional diabetes treatments. To exemplify this, reports of minor hypoglycaemia with liraglutide 1.2 mg and 1.8 mg were at placebo level and lower than glimepiride treatment in the LEAD-2 study: 0.03 and 0.09 events/patient/year (liraglutide 1.2 mg and 1.8 mg, respectively) *versus* 0.13 (placebo) and 1.23 (glimepiride) events/patient/year [26]. Furthermore, the event rate for minor hypoglycaemia was lower with liraglutide than with exenatide (1.93 *versus* 2.60 events/patient/year, respectively; *p* = 0.0131) [30].

**Figure 12:**
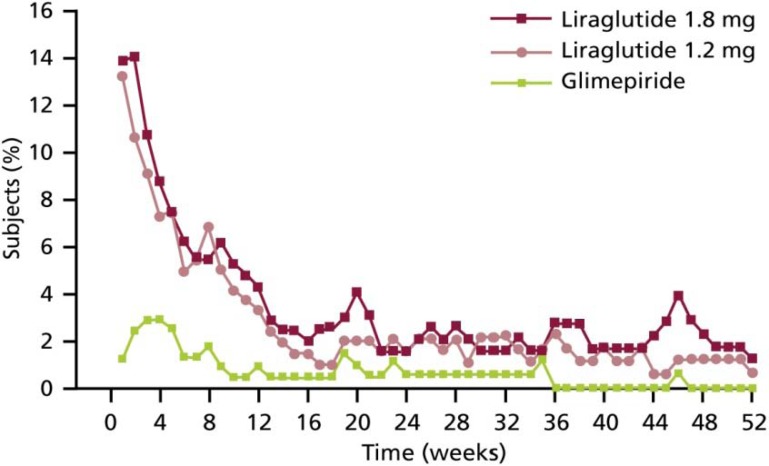
Proportion of Subjects with Nausea by Week and Treatment in LEAD-3 [27].

Overall, liraglutide is generally well tolerated, with most adverse events across the LEAD studies reported to be mild or moderate in severity and frequently gastrointestinal-related. Nausea was the most common adverse effect; it was reported by up to 40% of patients [25,26,27,28,29,30]. Nausea, however, was transient in nature and tended to dissipate after four weeks of treatment (Figure 12). The incidence of nausea was similar to exenatide initially, but it was less persistent with liraglutide [30]. Of note, very few adverse effects were serious and withdrawals were rare; the majority of these withdrawals were due to nausea [25,26,27,28,29,30]. 

There have been very few cases of acute pancreatitis reported during the phase 3 trials with liraglutide; a total of seven cases were reported, six of which were in liraglutide-treated patients and one in the comparator group [25,26,27,28,29,34]. No episodes of acute pancreatitis were reported with either exenatide or liraglutide in the head-to-head trial [30]. This risk rate was not higher than the background populations investigated. Indeed, patients with type 2 diabetes have a 2.8-fold higher risk than the general population of developing pancreatitis [35]. The increased incidence of pancreatitis is thought to be a class effect since it has also been observed in association with exenatide use; some researchers suggest that GLP-1 receptor activation through exogenous GLP-1 may increase pancreatic mass and/or modulate a genetic effect [36].However, these findings have been shown in animals only, and further research is needed. At this stage, liraglutide use is to be discontinued in patients exhibiting the signs of acute pancreatitis (severe abdominal pain) [34].

High sequence identity with native GLP-1 leads to low antibody formation associated with liraglutide use, and the LEAD studies showed no indication that anti-liraglutide antibodies had any impact on indices of efficacy or safety [25,26,27,28,29,30].

Concerns following an observation of increased thyroid conditions (including calcitonin, goitre and thyroid) in patients with pre-existing thyroid disease [34] suggest that liraglutide should not be used in patients with such concomitant conditions, and it would seem to be appropriate to discontinue liraglutide should such an illness emerge following treatment initiation.

## 7. How Liraglutide Can Be Used in Clinical Practice

This review focuses primarily on clinical trial research as the most stringent source of current data concerning the efficacy and safety of liraglutide; however, following its recent approval by both the FDA and the EMEA, it is to be anticipated that further research and material concerning liraglutide use in clinical practice will become available. This section considers liraglutide use in the clinic in the light of current advised practice.

## 8. When Should Liraglutide Be Used in the Treatment Paradigm?

**Figure 13:**
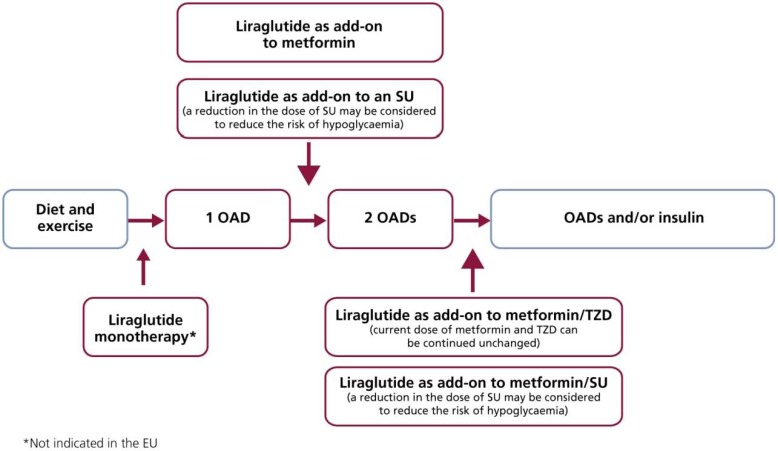
Liraglutide Potential Use in Clinical Practice, as Evidenced by the LEAD Programme.

The LEAD programme has demonstrated that liraglutide is effective and well tolerated as a starting therapy (monotherapy) or as an add-on therapy for subjects at a later stage of disease progression. Based on results from the LEAD programme, it is possible to recommend potential applications for liraglutide use in clinical practice (Figure 13), and in these studies liraglutide has been demonstrated as an effective therapeutic intervention regardless of when, or with what, it has been used. 

Although the trial data suggest a broad application, experience of liraglutide in clinical practice is limited. The best possible stage of treatment intervention is still speculative. Given a potential effect, if as yet unsubstantiated, on β-cell preservation [11,12,31], it seems possible that earlier use will extend the endogenous insulin function of patients, and perhaps slow progression of the disease. A meta-analysis of clinical data showed that reductions in HbA_1c_ and weight were achieved within eight weeks of liraglutide therapy, from which the authors concluded that adding liraglutide to an existing OAD regimen early in therapy may benefit patients [37]. Substantial reductions in SBP and FPG were also observed [37]. In terms of durability of use, that is, how long liraglutide can maintain good glycaemic control before an additional and/or alternative agent is needed, the LEAD studies generally showed that HbA_1c_ levels were sustained for the 26-week trial period [25,26,28,29,30]. The LEAD-3 study, exploring liraglutide as monotherapy, lasted for 52 weeks [27], suggesting that liraglutide’s effects may be sustained in longer-term treatment. However, given the individuality of each patient, it seems likely that the continued monitoring and assessment characteristic of treatment management in type 2 diabetes will be necessary to evaluate the durability of this new drug.

One question of particular importance concerns the relationship of liraglutide to insulin use; patients with type 2 diabetes will usually require exogenous insulin as their endogenous response fails, but barriers to insulin use are substantial. Concerns regarding weight gain and hypoglycaemia, two commonly feared side effects to insulin use [38], may mean that insulin is introduced late into the regimen once glycaemic control has started to fail. A comparison of liraglutide and insulin glargine in the LEAD-5 study demonstrated superior efficacy of liraglutide, suggesting that the drug could also be used as an alternative OAD to insulin [29]. The progressive nature of type 2 diabetes may make, insulin use inevitable for many patients, however,liraglutide may enable physicians to delay this treatment evolution, while maintaining good glycaemic control. Liraglutide may be a more helpful adjunct to metformin than SUs, given that the side effects of weight gain and hypoglycaemia are significantly lower; this, in turn, may support patient adherence and acceptance of therapy. Equally, adding liraglutide to an existing SU-based regimen may ameliorate some of the negative side effects.

Liraglutide has been approved by the European Medicines Agency (EMEA) and is currently implicated as a second- and third-line treatment option for type 2 diabetes [34]. 

*Second-line treatment:* Liraglutide in combination with metformin or SU, in patients with insufficient glycaemic control despite maximal tolerated dose of monotherapy with metformin or an SU. 

*Third-line treatment:* Liraglutide in combination with metformin and an SU or metformin and a TZD in patients with insufficient glycaemic control despite dual therapy.

Most recently, the FDA has approved liraglutide use as second- or third-line adjunct therapy, but also, significantly, as a monotherapy.

There are typically three steps in the current treatment guidelines to adhere to. According to the ADA/European Association for the Study of Diabetes (EASD) guidelines, at the onset of type 2 diabetes (step 1), lifestyle intervention and metformin is initiated [39]. Traditionally, ‘well-validated’ basal insulin is considered when these patients fail to reach or sustain glycaemic targets (step 2), and further intensification of insulin is required (step 3). Currently, GLP-1 receptor agonists, including liraglutide, are recommended as second-line therapy by the ADA/EASD guidelines as a ‘less well-validated’ treatment [39]. 

In a statement, the American Association of Clinical Endocrinologists (AACE) guidelines recommend the use of incretin mimetics (GLP-1 receptor agonists) as second-line therapy, followed by DPP-4 inhibitors, glinides, or sulphonylureas (SUs) with metformin remaining the cornerstone of dual therapy. Indeed, dual therapy is recommended if the HbA_1c_ ranged between 7.6% and 9.0% [40]. The panel recognised the lower risk of hypoglycaemia associated with GLP-1 receptor agonists and DPP-4 inhibitors compared with glinides and SUs, and were favoured over DPP-4 inhibitors as they demonstrated greater postprandial glucose lowering and substantial weight loss. For patients requiring triple therapy (*e.g.*, HbA_1c_ > 9.0%), a GLP-1 receptor agonist is the second preferred agent [40]. The National Institute for Clinical Excellence (NICE) also recommends GLP-1 receptor agonists use in combination with metformin and an SU as a third-line treatment option [41].

## 9. Conclusions

The role of GLP-1 receptor agonists as a treatment option for type 2 diabetes is rapidly evolving. Liraglutide, the first human GLP-1 analogue, has the potential to overcome the shortcomings of conventional therapies and address the current unmet needs of patients with type 2 diabetes. 

With its once-daily administration, liraglutide has demonstrated superior clinical efficacy and a favourable safety profile in recent clinical trials *versus* standard therapies. Indeed, glycaemic control improved substantially and was maintained throughout the trial period with liraglutide. Furthermore, reports of hypoglycaemia were rare. Liraglutide has also been found to promote and maintain weight loss and reduce SBP, which, in turn, may reduce the risk of CV disease. Moreover, reductions in SBP appear not to be influenced by body weight change. Interestingly, there is an indication that liraglutide may have a positive effect not only on β-cell function but also on β-cell mass, which implies potential long-term benefits. Compared with exenatide, liraglutide offers similar efficacy, but with once-daily dosing, which may be of importance to patients. Similarly, given the greater sequence identity between liraglutide and native GLP-1, fewer antibodies are formed, and this may account for improved tolerance in terms of side effects. For patients with type 2 diabetes, liraglutide offers a new and flexible treatment alternative to standard therapies that may help overcome the unmet needs of current therapies.
